# The relationship between upper airway parameters and COVID-19 symptom severity in adolescents

**DOI:** 10.3389/froh.2024.1458368

**Published:** 2024-11-14

**Authors:** Tianjing Du, Juan Wang, Peter Mei, Dongning Li, Jiamin Zhao, Jianglin Zhou, Jun Wang, Yifei Xu, Kun Qi

**Affiliations:** ^1^ Key Laboratory of Shaanxi Province for Craniofacial Precision Medicine Research, College of Stomatology, Xi'an Jiaotong University, Xi'an, China; ^2^ Discipline of Orthodontics, Department of Oral Sciences, Faculty of Dentistry, University of Otago, Dunedin, New Zealand; ^3^ Department of Otorhinolaryngology Head and Neck Surgery, Shaanxi Provincial People's Hospital, Xi'an, China; ^4^ Department of Oral Anatomy and Physiology and TMD, School of Stomatology, The Fourth Military Medical University, Xi'an, China

**Keywords:** COVID-19, upper airway, questionnaire, CBCT, symptom assessment, orthodontic treatment, respiratory diseases

## Abstract

**Background:**

COVID-19 is a respiratory disease, and its symptoms may be affected by the upper airways of adolescents.

**Objective:**

To investigate the effect of parameters of adolescents’ upper airways on COVID-19 symptom severity.

**Methods:**

This retrospective study was performed from January to March 2022 at the Hospital of Stomatology, Xi’an Jiaotong University, Xi’an, China. The inclusion criteria were patients who started orthodontic treatment for the first time, who experienced initial onset of laboratory-confirmed COVID-19, and who received two intramuscular doses of the SARS-CoV-2 vaccine. Participants’ COVID-19 symptom severity was recorded by a questionnaire including seven different dimensions. The three-dimensional parameters of the upper airway were obtained by cone beam computed tomography (CBCT) and measured by Dolphin Imaging software by blinded orthodontic investigators. The correlation between COVID-19 symptom severity and three-dimensional upper airway parameters was analyzed.

**Results:**

64 males (46.4%) and 74 females (53.6%) were included in the study, with the median age of 9.5 years. The severity score of dimension 3 (headache, muscle pain, fatigue, shortness of breath, diarrhea and smell affects) showed a linear relationship with age. Spearman's rank correlation showed that the severity score of dimension 1 (nasal symptoms) was negatively correlated with nasal volume (*r* = −0.325). The severity score of dimension 6 was negatively correlated with the height of the nasopharynx (*r* = −0.325) and positively correlated with the horizontal-to-vertical ratio of the oropharynx (*r* = 0.385).

**Conclusions and relevance:**

The COVID-19 symptom severity was aggravated with the increase of age. Nasal and throat pain and dry mouth was negatively correlated with nasal volume and nasopharyngeal height. The COVID-19 symptom severity among individuals is relavant to age and upper airway.

## 1 Introduction

The coronavirus disease 2019 (COVID-19) pandemic, due to the novel severe acute respiratory syndrome coronavirus 2 (SARS-CoV-2), has caused a worldwide sudden. As of July 1, 2020, SARS-CoV-2 has affected more than 200 countries, resulting in more than 10 million identified cases with 508,000 confirmed deaths (1) and COVID-19 continues to circulate (2).

COVID-19 has a spectrum of asymptomatic infection to mild/moderate pneumonia and/or severe respiratory syndrome with fatal outcomes (3). The primary symptoms of COVID-19 include fever, cough, sore throat, and fatigue, and some patients experience nasal symptoms such as congestion and runny nose, as well as gastrointestinal symptoms such as diarrhea and vomiting (4–7). Neurological symptoms, including headache, dizziness, and loss of smell and taste, have also been reported (8). Oral manifestations are common in COVID-19 patients and mainly include taste disorders, mucosal lesions, dry mouth, and oral ulcers (9). A minority of patients with severe COVID-19 may develop critical complications, such as acute respiratory distress syndrome (ARDS) and multiorgan failure (10), leading to death and other adverse outcomes. Several risk factors for poor outcomes, including age, obesity, and respiratory and cardiovascular diseases, have been identified (11, 12). Adolescents with COVID-19 typically exhibit asymptomatic or mild cases (13), and COVID-19 has a lower incidence and severity in adolescents than in adults; this may be due to healthier respiratory systems, different respiratory receptor expression, and possibly immune systems that offer protection against SARS-CoV-2 infection, although the exact reasons remain to be explored (14). Therefore, it is of clinical significance to explore the reasons leading to different severity of COVID-19 symptoms (15). At the same time, it will also provide some new ideas in the study of influencing factors that cause different symptoms of respiratory diseases, which is conducive to the management and control of respiratory diseases in clinic.

COVID-19 is a respiratory disease; hence, there is a possible relationship between symptom severity and upper airway parameters. Recent studies have indicated that in adults with COVID-19, numerous parameters, such as lower facial height (LFH), vertical airway length (VAL), and position of the hyoid bone relative to the mandible, may be associated with the severity of COVID-19 symptoms (16). However, the correlations between three-dimensional parameters of adolescent upper airway parameters and the COVID-19 symptom severity remain unclear.

The aim of this study was to investigate the correlation between COVID-19 symptom severity and three-dimensional (3D) upper airway morphologies in adolescents. The findings of this study can provide a reference for the prediction of COVID-19 prognoses in adolescents.

## 2 Methods

### 2.1 Study design and participants

This retrospective study was performed at the Hospital of Stomatology, Xi’an Jiaotong University, in Xi’an, China, between January and March 2022. This study was approved by the Ethics Committee of Xi’an Jiao-tong University (2023-XJKQIEC-QT-0020-002).

The inclusion criteria were as follows: (1) patients who started orthodontic treatment for the first time between January and March 2022 at the Hospital of Stomatology, Xi’an Jiaotong University; (2) patients with initial onset of laboratory-confirmed COVID-19; and (3) patients who received two intramuscular doses of the SARS-CoV-2 vaccine. The exclusion criteria were as follows: (1) patients who had suffered from respiratory infections other than COVID-19 in the past three months; (2) patients who suffered from systemic disease, craniofacial deformities or other developmental disorders; and (3) patients who previously underwent any orthodontic treatment.

### 2.2 Procedures

#### 2.2.1 Symptom severity investigation

The participants were interviewed in person by respiratory medicine specialists and asked to complete a series of questionnaires on demographic characteristics (age, sex, education), nasal obstruction symptom evaluation (NOSE) scores (17) and COVID-19 symptom severity. Set options for each question in the questionnaire and assign different points to questions. The reliability and validity of this questionnaire were evaluated by exploratory factor analysis (EFA) (18, 19), which divides the dimensions of the questionnaire, was used to determine the number of factors retained by the questionnaire. Informed consent was obtained from all study participants.

#### 2.2.2 Airway measurements

All CBCT scans were performed using the same procedure with similar cone beam equipment (i-CAT, Imaging Sciences International, Hatfield, PA, USA) (120 kV, 5 mA, 14 *17 cm FOV, 0.4 mm voxel, and scan time of 8.9 s). Patients were asked to sit erect and hold their breath at the end of expiration, with their head in a natural posture, their jaw in maximal intercuspation, and not to swallow. All digital image data were recorded in Digital Imaging and Communications in Medicine (DICOM) format. The 3D airway parameters were measured by Dolphin Imaging software version 11.7. (Dolphin Imaging & Management Solutions, Chatsworth, CA, USA).

CBCT was analyzed by blinded orthodontic investigators with more than ten years of clinical experience. The interrater consistency test (kappa >0.8) showed good reliability. CBCT parameters included the airway volume, airway length, minimal airway area, anteroposterior (A-P) distance of the minimal airway area, and lateral distances of the minimal airway area of the nasopharynx, oropharynx and laryngopharynx.

**Figure 1:**
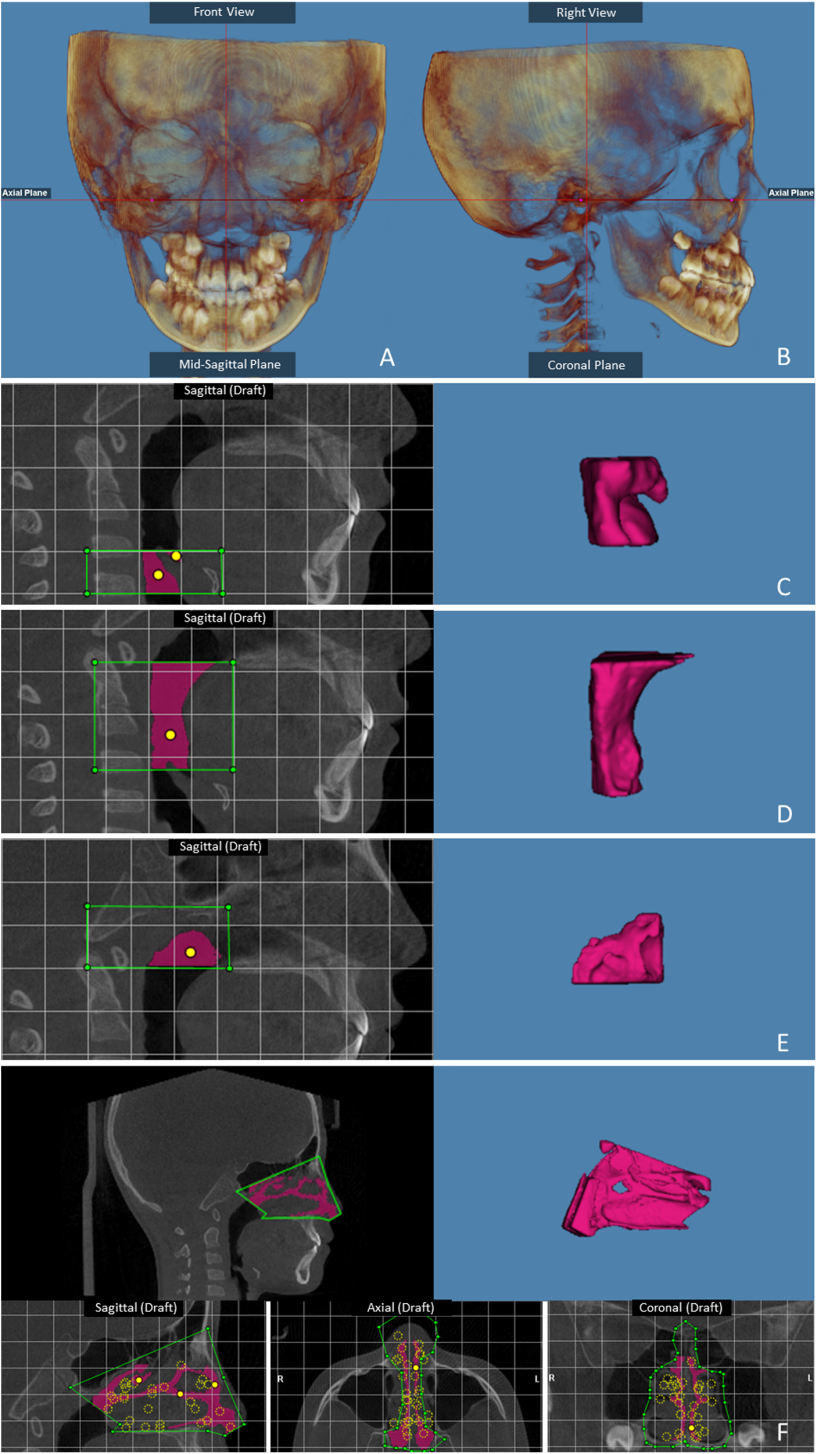
Skull orientation: **(A)** frontal view; **(B)** right sagittal view. The landmarks for the airway: **(C)** Laryngeal airway volume; **(D)** Oropharyngeal airway volume; **(E)** Nasopharyngeal airway volume. **(F)** The nasal landmarks.

3D volume images of the skull were reoriented. The axial plane was used to orient the skull on the Frankfort horizontal plane. The horizontal reference line was produced using the porion and right orbitale in the right sagittal view. In the frontal view, the horizontal reference line was established by the right and left orbitales. The vertical reference line was established through the nasion and the anterior nasal spine (20). This established a reference plane so that all images could be standardized to this position before airway measurement. Then, the best para-sagittal view of the airway that allowed for clear visualization of the posterior nasal spine and of the second cervical vertebra was identified. The patient airway was identified by viewing sequential slices of the volume and placing seed points in the voids on the image that represent the patient airway. Based on the seed points, the software automatically selected the adjacent empty areas and identified the patient airway. The airway was divided into 2 spaces: the lower space, corresponding to the oropharynx and velopharynx, and the upper space, corresponding to the nasopharynx. The most inferior boundary of the lower space of the airway was determined to be the plane drawn through the most anterior-inferior point of the second cervical vertebra and parallel to the Frankfort plane. The most superior boundary of the lower space of the airway and the most inferior boundary of the upper space were determined to be the plane connecting the posterior nasal spine and the most superior point of the anterior arch of the atlas. The posterior-superior-anterior boundaries of the upper space of the airway were defined by lines connecting the atlas, basion, spheno-occipital synchondrosis, superior and anterior edges of the vomer bone, and posterior nasal spine. Once the portion of the field of interest was selected, the software automatically calculated the pharyngeal volume in cubic millimeters, the cross-sectional area in square millimeters and the minimal airway area (21, 22) (Figure 1).

#### 2.2.3 Statistical analysis

All the data were analyzed using SPSS 18.0. After the normality test of data, the following statistical methods were selected. The demographic characteristics and symptom scale scores of COVID-19 patients are presented as interquartile range (IQR) for continuous variables. For the comparison of COVID-19 symptom severity between people with different sex and education, we used the Mann‒Whitney *U*-test where appropriate. To explore the relationship of age and COVID-19 symptom severity, regression models were used. Spearman rank correlation was selected for the correlation between COVID-19 symptom severity and airway parameters. All tests were two-sided. A *p*-value less than 0.05 was considered to indicate statistical significance.

## 3 Results

### 3.1 Reliability and validity of the questionnaire

The reliability alpha of the questionnaire was 0.926 (29 items). The validity of the questionnaire was examined by exploratory factor analysis. KMO > 0.5 and Bartlett's sphericity test *P* < 0.001 suggested that there was enough variability in the items to perform EFA. Through exploratory factor analysis, the 26 questions on the COVID-19 symptom severity in the questionnaire were divided into seven dimensions, and the factor coefficients for each dimension were greater than 0.5, indicating that the dimensions were well divided. According to the results of the dimension division, dimension 1 mainly described nasal symptoms, dimension 2 mainly described cough symptoms, dimension 5 mainly described fever symptoms, and the other dimensions contained scattered symptoms, including those related to the pharynx, mouth, pain, smell, nausea and other aspects (Table 1).

**Table 1:** Results of the dimension division in the questionnaire.

Number	Questions
Dimension 1	
1	Stuffy or blocked nose
2	Poor nasal ventilation
3	Hard to breathe through the nose
4	Unable to breathe smoothly through the nose during exercise or exertion
5	Nasal congestion interferes with sleep
6	Duration of nasal congestion
Dimension 2	
1	The extent to which coughing affects daily life
2	How often cough occurs
3	The extent to which coughing affects sleep at night
4	Easily fatigued by coughing
5	Duration of cough
6	Cough more frequently when they encounter dust, smell irritants, and inhale cold air
Dimension 3	
1	Duration of headache
2	Degree of muscle pain
3	Duration of fatigue
4	Duration of shortness of breath
5	Duration of muscle pain
6	Duration of diarrhea
7	Duration of smell affects
8	Degree of head pain
Dimension 4	
1	Duration of nausea
2	Duration of mouth pain
3	Duration of periodontal symptoms
Dimension 5	
1	Heating temperature
2	Duration of heating
Dimension 6	
1	Duration of sore throat
2	Duration of dry mouth
Dimension 7	
1	Degree of pharyngeal pain
2	Duration of oral disease

Extraction method: Principal component analysis.

### 3.2 Description of demographic characteristics and COVID-19 symptom severity

The current study included 138 participants, including 64 males (46.4%) and 74 females (53.6%). The median age of participants was 9.5 years. CBCT imaging data for the past six months were available for 42 participants (17 males and 25 females).

As shown in Table 2, the median score for dimension 5 (fever symptoms) was the highest (2.333), and the median score for dimension 4 (nausea, mouth pain and periodontal symptoms) and 7 (pharyngeal pain and oral disease) was the lowest. The severity of dimension 3(*r*^2^ = 0.171) had a linear relationship with age, and was increased with increasing age (Table 3). The linear regression coefficient had statistical significance, which means that age could be used as a predictor of the severity of symptoms in this dimension. There was no significant difference in the COVID-19 symptom severity between males and females.

**Table 2:** Severity scale of the participants in each dimension.

		Dimension 1	Dimension 2	Dimension 3	Dimension 4	Dimension 5	Dimension 6	Dimension 7
Median		0.778	0.556	0.381	0.000	2.333	0.667	0.000
Std. deviation		0.802	0.878	0.789	0.447	0.840	1.119	0.604
Minimum		0.000	0.000	0.000	0.000	0.000	0.000	0.000
Maximum		4.000	3.500	3.700	2.670	3.500	4.000	4.000
Percentile	25	0.000	0.000	0.000	0.000	1.667	0.000	0.000
	75	1.333	1.292	1.129	0.000	2.833	2.000	0.000

**Table 3:** Linear regression coefficient analysis between the severity of dimension 3 and participant's age^a^.

	Constant	B	R Square	Sig.
Dimension 3	8.497	1.837	0.171	<0.001*

^a^ Dependent variable: age.

* The correlation is significant when the confidence (2-tailed) is 0.05.

### 3.3 Correlations between COVID-19 symptom severity and upper airway parameters

The symptom severity on dimension 1 was negatively correlated with nasal volume (*r* = −0.325), indicating that nasal symptoms of COVID-19 decreased with increasing nasal volume (Table 4). The severity score of dimension 6 was negatively correlated with the height of the nasopharynx (*r* = −0.325) and positively correlated with the horizontal-to-vertical ratio of the oropharynx (*r* = 0.385). In other words, as the height of the nasopharynx increased, the shape of the oropharynx became increasingly narrower and longer, and the severity of pharyngeal pain and dry mouth decreased in patients with COVID-19.

**Table 4:** Correlation coefficient between airway parameters and severity scale of each dimension.

	Dimension 1	Dimension 2	Dimension 3	Dimension 4	Dimension 5	Dimension 6	Dimension 7
Nasal volumes	−.325^a^	−.171	.335^a^	.009	.073	−.038	−.085
Nasopharyngeal volumes	−.231	.004	.243	.022	.093	.013	−.081
Nasopharyngeal heights	−.014	.107	−.122	.208	.133	−.325^a^	−.073
Minimum areas of nasopharynx	−.259	−.023	.242	−.032	.015	.232	−.127
Lateral/A-P distances of nasopharynx	.193	−.302	−.001	−.030	−.046	−.304	.221
Lateral distances of nasopharynx	−.205	−.260	.220	−.084	.012	−.020	−.129
Anteroposterior distances of nasopharynx	−.258	.183	.104	−.051	.042	.278	−.216
Oropharyngeal volumes	−.129	.252	.331^a^	.081	−.113	.006	−.122
Oropharyngeal heights	−.104	.239	.513^b^	.051	−.018	.125	−.376
Minimum oropharyngeal areas	−.031	.292	.260	.044	−.090	−.077	−.074
Lateral/A-P distances of oropharynx	−.202	−.176	.331^a^	.185	−.132	.385^a^	−.097
Lateral distances of oropharynx	−.209	.006	.274	.060	−.163	.187	−.187
Anteroposterior (A-P) distances of oropharynx	.057	.250	−.025	−.160	−.148	−.173	.077
Laryngeal volumes	−.173	.037	.523^b^	−.002	.064	.115	−.285
Laryngeal heights	−.058	−.045	.392^a^	−.047	−.049	.167	−.186
Minimum laryngeal areas	−.092	.146	.364^a^	.088	.043	.002	−.178
Lateral/A-P distances of larynx	.074	−.282	.014	.048	.066	−.042	.097
Lateral distances of larynx	−.075	−.171	.204	.041	.090	.030	−.036
Anteroposterior (A-P) distances of larynx	−.181	.179	.110	−.016	.076	.103	−.157
PNS cross-sectional areas	−.101	.139	.340^a^	.288	.131	.101	−.182
Lateral distances of PNS	−.205	.157	.406^b^	.095	−.026	.077	−.262
Anteroposterior (A-P) distances of PNS	−.083	.189	.341^a^	.062	.227	.183	−.163
Lateral/A-P distances of PNS	−.136	−.112	−.127	.068	−.271	−.125	−.057
C3 cross-sectional areas	−.060	.327^a^	.298	.074	.024	−.129	−.145
Lateral distances of C3	−.085	.090	.438^b^	.070	.105	.019	−.257
Anteroposterior (A-P) distances of C3	−.167	.257	.051	.076	−.150	.037	−.063
C4 cross-sectional areas	.033	.045	.412^b^	.038	.012	.022	−.228
Lateral distances of C4	.009	−.155	.196	.049	.182	.023	−.041
Anteroposterior (A-P) distances of C4	−.021	.171	.248	−.013	.076	.141	−.181

^a^ The correlation is significant when the confidence (2-tailed) is 0.05.

^b^ The correlation is significant when the confidence (2-tailed) is 0.01.

The severity of dimension 3 was positively correlated with nasal volume (*r* = 0.335), oropharyngeal volume (*r* = 0.331), oropharyngeal height (*r* = 0.513), the horizontal-to-vertical ratio of the oropharynx (*r* = 0.331), laryngeal volume (*r* = 0.523), laryngeal height (*r* = 0.392), the minimum cross-sectional area of the larynx (*r* = 0.364), the cross-sectional area of the PNS (*r* = 0.340), the transverse diameter of the PNS (*r* = 0.406), and the longitudinal diameter of the PNS (*r* = 0.341), indicating that as scales of these airway parameters increased, the symptoms of dimension 3 worsened.

## 4 Discussion

To our knowledge, this is the first study on the correlation between COVID-19 symptom severity and 3D upper airway parameters. The findings of the present study suggest that headache, muscle pain, fatigue, shortness of breath and diarrhea worsen with increasing age. The severity of nasal and throat pain and dry mouth were negatively correlated with nasal volume and nasopharyngeal height. COVID-19 symptom severity was negatively correlated with parameters of the nasal cavity and nasopharynx.

Research on infectious diseases has been considered difficult because many infections and their health outcomes present spatiotemporal and population heterogeneity (23). The risk of infection among participants in infectious disease studies also differs due to factors such as previous infection history or antibodies obtained by vaccination, thus affecting the reliability of the study results. The current study recruited participants who were infected with COVID-19 for the first time and who had received two doses of the COVID-19 vaccine, which eliminates the influence of antibodies generated due to previous infection history. The data obtained on the severity of symptoms after COVID-19 better reflect reality and provide baseline data for future studies on COVID-19 reinfection. As there is no official or authoritative questionnaire on COVID-19 symptoms, this study integrated numerous mature scales on respiratory disease symptoms and comprehensively evaluated the severity and duration of symptoms in the nose, pharynx, mouth, head, and other areas and pain and fever.

The results of the present study showed that some symptoms, such as headache, muscle pain, fatigue, shortness of breath and diarrhea, worsened with increasing age, which was consistent with the findings of previous studies (24–26). Dimensions 1, 3, and 6, which included the severity of nasal symptoms, throat pain and dry mouth, were negatively correlated with nasal volume and nasopharyngeal height. It is speculated that due to the large volume of the nasal cavity and the nasopharynx height, more moist air enters from nasal cavity, which reduces the severity of dry mouth and throat pain. On the other hand, the original large volume of the nasal cavity is less affected by mucosal swelling after viral infection, so that nasal symptoms such as nasal congestion will be alleviated (27). The human coronaviruses (HCoVs) are respiratory diseases that also have respiratory symptoms (28). This can be explained by the antigenic similarity (as well as antibodies or immunity to those antigens) between COVID-19 and other HCoVs, which may cause cross-protection and similar symptoms (29). Therefore, it is predicted that changes in parameters of upper airway also could alleviate symptoms of respiratory diseases, such as HCoVs. Other research has shown that orthopaedic treatments can significantly increase, in the short-term, the dimensions and/or volume of the upper airways in children or young adolescents with growth potential (30). The size of the nasopharyngeal airway might be connected to the maxillary width because the maxilla comprises most of the lateral walls of the nasal cavity (20), and rapid maxillary expansion (RME) significantly increases the nasal airway volume but has no effect on the pharyngeal airway volume (24, 31). The direct relationship between the extension of maxillomandibular advancement and an increased volume in the upper airways is also well established (32). Therefore, it reflects another major advantage of early orthodontic treatment and adenoidectomy in addition to promoting normal craniofacial development. These findings suggest that airway assessment in orthodontic treatment may be of great significance for the clinical management of respiratory diseases.

There are limitations in the present study. The questionnaire data were based on the patients reports, which were subjective. The results of this study showed that the severity of dimension 3 (including headache, muscle pain, fatigue, shortness of breath, diarrhea and olfaction symptoms) was positively correlated with the oropharynx and laryngeal volume. According to the questionnaire factor analysis, symptoms in dimension 3 were scattered, and the cross-influence among symptoms made the relationship between the study results and reality unclear. Therefore, to eliminate the influence of confounding factors and obtain a more realistic conclusion, future studies on the separate symptoms are needed.

## 5 Conclusion

COVID-19 symptom severity was negatively correlated with parameters of the nasal cavity and nasopharynx. Patients with upper airway agumentation through orthopaedic treatments tend to have mild respiratory disease symptoms.

## Data Availability

The raw data supporting the conclusions of this article will be made available by the authors, without undue reservation.

## References

[1] Wiersinga WJ Rhodes A Cheng AC Peacock SJ Prescott HC. Pathophysiology, transmission, diagnosis, and treatment of coronavirus disease 2019 (COVID-19): a review. JAMA. (2020) 324(8):782–93. 10.1001/jama.2020.12839 32648899

[2] Kontoghiorghes GJ Kolnagou A Kontoghiorghe CN. Post COVID-19 reflections and questions: how prepared are we for the next pandemic? Int J Mol Sci. (2024) 25(2):5. 10.3390/ijms25020859 PMC11326220 38255933

[3] The Novel Coronavirus Pneumonia Emergency Response Epidemiology Team. The epidemiological characteristics of an outbreak of 2019 novel coronavirus diseases (COVID-19)—China, 2020. China CDC Weekly. (2020) 2(8):113–22. 10.46234/ccdcw2020.032 34594836 PMC8392929

[4] Alizadehsani R Alizadeh Sani Z Behjati M Roshanzamir Z Hussain S Abedini N Risk factors prediction, clinical outcomes, and mortality in COVID-19 patients. J Med Virol. (2021) 93(4):2307–20. 10.1002/jmv.26699 33247599 PMC7753243

[5] Huang C Wang Y Li X. Clinical features of patients infected with 2019 novel coronavirus in Wuhan, China (vol 395, pg 497, 2020). Lancet. (2020) 395(10223):496. 10.1016/S0140-6736(20)30183-5 PMC7159299 31986264

[6] Li J Huang DQ Zou B Yang H Hui WZ Rui F Epidemiology of COVID-19: a systematic review and meta-analysis of clinical characteristics, risk factors, and outcomes. J Med Virol. (2021) 93(3):1449–58. 10.1002/jmv.26424 32790106 PMC7436673

[7] Qaisieh R Al-Tamimi M El-Hammuri N Shalabi M Kilani MM Taha H Clinical, laboratory, and imaging features of COVID-19 in a cohort of patients: cross-sectional comparative study. JMIR Public Health Surveill. (2021) 7(9):19. 10.2196/28005 PMC8457339 34081600

[8] Mao L Jin HJ Wang MD Hu Y Chen SC He QW Neurologic manifestations of hospitalized patients with coronavirus disease 2019 in Wuhan, China. JAMA Neurol. (2020) 77(6):683–90. 10.1001/jamaneurol.2020.1127 32275288 PMC7149362

[9] Sharma P Malik S Wadhwan V Palakshappa SG Singh R. Prevalence of oral manifestations in COVID-19: a systematic review. Rev Med Virol. (2022) 32(6):18. 10.1002/rmv.2345 PMC9111150 35271738

[10] Zhou F Yu T Du R. Clinical course and risk factors for mortality of adult inpatients with COVID-19 in Wuhan, China: a retrospective cohort study (vol 395, pg 1054, 2020). Lancet. (2020) 395(10229):1038. 10.1016/S0140-6736(20)30566-3 PMC7270627 32171076

[11] Chenchula S Vidyasagar K Pathan S Sharma S Chavan MR Bhagavathula AS Global prevalence and effect of comorbidities and smoking status on severity and mortality of COVID-19 in association with age and gender: a systematic review, meta-analysis and meta-regression. Sci Rep. (2023) 13(1):16. 10.1038/s41598-023-33314-9 37076543 PMC10115382

[12] O'Driscoll M Ribeiro Dos Santos G Wang L Cummings DAT Azman AS Paireau J Age-specific mortality and immunity patterns of SARS-CoV-2. Nature. (2021) 590(7844):140–5. 10.1038/s41586-020-2918-0 33137809

[13] Cui X Zhao Z Zhang T Guo W Guo W Zheng J A systematic review and meta-analysis of children with coronavirus disease 2019 (COVID-19). J Med Virol. (2021) 93(2):1057–69. 10.1002/jmv.26398 32761898 PMC7436402

[14] Sinaei R Pezeshki S Parvaresh S Sinaei R. Why COVID-19 is less frequent and severe in children: a narrative review. World J Pediatr. (2021) 17(1):10–20. 10.1007/s12519-020-00392-y 32978651 PMC7518650

[15] Ballering AV van Zon SKR Olde Hartman TC Rosmalen JGM, Lifelines Corona Research Initiative. Persistence of somatic symptoms after COVID-19 in The Netherlands: an observational cohort study. Lancet. (2022) 400(10350):452–61. 10.1016/S0140-6736(22)01214-4 35934007 PMC9352274

[16] Al Maaitah EF Al-Musfir TM Al Jawad FA Alhashimi N Abu Alhaija ES. Upper airway dimensions and the skeletal parameters in orthodontic patients who developed moderate-severe COVID-19 symptoms during the pandemic. Dent Med Probl. (2023) 60(1):13–22. 10.17219/dmp/157457 36921256

[17] Lipan MJ Most SP. Development of a severity classification system for subjective nasal obstruction. JAMA Facial Plast Surg. (2013) 15(5):358–61. 10.1001/jamafacial.2013.344 23846399

[18] Gaskin CJ Happell B. On exploratory factor analysis: a review of recent evidence, an assessment of current practice, and recommendations for future use. Int J Nurs Stud. (2014) 51(3):511–21. 10.1016/j.ijnurstu.2013.10.005 24183474

[19] Sharkey CM Perez MN Bakula DM Grant DM Mullins LL. Exploratory factor analysis of the Mishel uncertainty in illness scale among adolescents and young adults with chronic medical conditions. J Pediatr Health Care. (2019) 33(2):186–94. 10.1016/j.pedhc.2018.08.002 30177225

[20] Bertoz APD Souki BQ Lions R Webber SAT Bigliazzi R Oliveira PM Three-dimensional airway changes after adenotonsillectomy in children with obstructive apnea: do expectations meet reality? Am J Orthod Dentofac Orthop. (2019) 155(6):791–800. 10.1016/j.ajodo.2018.06.019 31153499

[21] Hsu WC Kang KT Yao CCJ Chou CH Weng WC Lee PL Evaluation of upper airway in children with obstructive sleep apnea using cone-beam computed tomography. Laryngoscope. (2021) 131(3):680–5. 10.1002/lary.28863 33070361

[22] Jia P Dong WH Yang SJ Zhan ZC Tu L Lai SJ. Spatial lifecourse epidemiology and infectious disease research. Trends Parasitol. (2020) 36(3):235–8. 10.1016/j.pt.2019.12.012 32044243 PMC7172117

[23] Incu I Davioiu AM Chindri S Pleca D. Pathogenesis of SARS-CoV-2 infection in humans. Pediatru Ro. (2020) 2(58):10. 10.26416/Pedi.58.2.2020.3572

[24] Abdalla Y Brown L Sonnesen L. Effects of rapid maxillary expansion on upper airway volume: a three-dimensional cone-beam computed tomography study. Angle Orthod. (2019) 89(6):917–23. 10.2319/101218-738.1 30942607 PMC8109167

[25] Loske J Rohmel J Lukassen S Stricker S Magalhaes VG Liebig J Pre-activated anti-viral innate immunity in the upper airways controls early SARS-CoV-2 infection in children. medRxiv. (2021). 10.1038/s41587-021-01037-9 34408314

[26] Xu JT. The role of upper airway morphology in apnea versus hypopnea predominant obstructive sleep apnea patients: an exploratory study. Br J Radiol. (2018) 91(1088):1. 10.1259/bjr.20180363 PMC6209479 29770707

[27] Ozdemir Akkus N İşçi KD. Etiology of narrow maxilla creating orthodontic and prosthetic treatment difficulties. Eur Rev Med Pharmacol Sci. (2023) 27(5 Suppl):75–9. 10.26355/eurrev_202310_34073 37869951

[28] McDon Ald E Pittet LF Barry SE Bonten M Campbell J Croda J Antecedent and persistent symptoms in COVID-19 and other respiratory illnesses: insights from prospectively collected data in the BRACE trial. The Journal of Iinfection. (2024) 89(5):106267. 10.1016/j.jinf.2024.106267 PMC11489119 39245151

[29] Şanlidağ Işbilen G Uysal AA Yiğit S Appak Ö Sipahi H Bozdayi G Do patients infected with human coronavirus before the COVID-19 pandemic have less risk of being infected with COVID-19? Turk J Med Sci. (2024) 54(4):761–5. 10.55730/1300-0144.5846 39295607 PMC11407371

[30] Bellon M Boutin F Haddad R Frapier L. Effectiveness of orthopaedic treatments on the enlargement of the upper airways: overview of systematic reviews. Int Orthod. (2023) 21(2):21. 10.1016/j.ortho.2023.100745 36871416

[31] Kavand G Lagravère M Kula K Stewart K Ghoneima A. Retrospective CBCT analysis of airway volume changes after bone-borne vs tooth-borne rapid maxillary expansion. Angle Orthod. (2019) 89(4):566–74. 10.2319/070818-507.1 30768911 PMC8117203

[32] Rana SS Kharbanda OP. Letter to editor on “efficiency of bimaxillary advancement surgery in increasing the volume of the upper airways: a systematic review of observational studies and meta-analysis”. Eur Arch Oto-Rhino-Laryn. (2017) 274(1):585. 10.1007/s00405-016-4118-y 27260165

